# The Built Environment Is a Microbial Wasteland

**DOI:** 10.1128/mSystems.00033-16

**Published:** 2016-04-19

**Authors:** Sean M. Gibbons

**Affiliations:** Department of Biological Engineering, Massachusetts Institute of Technology, Cambridge, Massachusetts, USA

**Keywords:** built environment, hygiene hypothesis, indoor, microbiome, office, outdoor

## Abstract

Humanity’s transition from the outdoor environment to the built environment (BE) has reduced our exposure to microbial diversity. The relative importance of factors that contribute to the composition of human-dominated BE microbial communities remains largely unknown. In their article in this issue, Chase and colleagues (J. Chase, J. Fouquier, M. Zare, D. L. Sonderegger, R. Knight, S. T. Kelley, J. Siegel, and J. G. Caporaso, mSystems 1(2):e00022-16, 2016, http://dx.doi.org/10.1128/mSystems.00022-16) present an office building study in which they controlled for environmental factors, geography, surface material, sampling location, and human interaction type. They found that surface location and geography were the strongest factors contributing to microbial community structure, while surface material had little effect. Even in the absence of direct human interaction, BE surfaces were composed of 25 to 30% human skin-associated taxa. The authors demonstrate how technical variation across sequencing runs is a major issue, especially in BE work, where the biomass is often low and the potential for PCR contaminants is high. Overall, the authors conclude that BE surfaces are desert-like environments where microbes passively accumulate.

## Abstract (precis)

Humanity’s transition from the outdoor environment to the built environment (BE) has reduced our exposure to microbial diversity. The relative importance of factors that contribute to the composition of human-dominated BE microbial communities remains largely unknown.

## COMMENTARY

**FIG 1:**
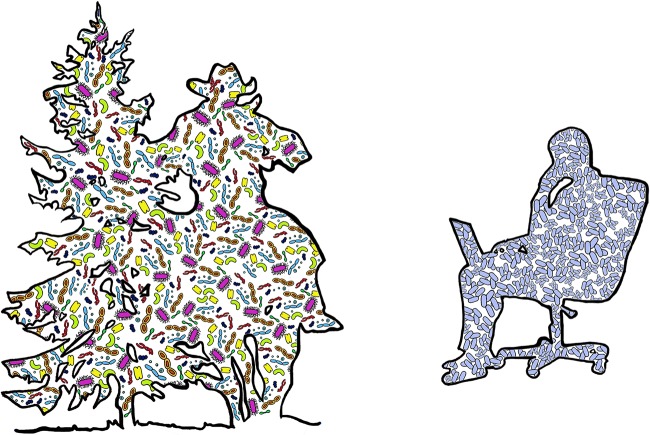
Microbial diversity in outdoor environments and BEs. On the left is the silhouette of a cowboy brushing past a pine tree while riding a horse. On the right is the silhouette of a person sitting in an office chair and working on a laptop. Blue microbes are human associated, while other colors represent nonhuman microbial diversity.

Multicellular organisms have evolved in close association with diverse microbial consortia. Certain members of these consortia are passed vertically from parent to offspring (1), while others are acquired from the environment (2). Modern humans have largely removed themselves from the outdoor environments in which we evolved (Fig. 1). We now spend most of our lives indoors—in our “built environment” (BE). This shift in lifestyle may have disrupted the successional processes whereby we acquire a fully functional adult microbiome (3–5). Microbial dysbioses have been linked to a number of developmental disorders and disease susceptibility (6–9). Indeed, the hygiene hypothesis posits that Western lifestyles are responsible for a wide array of autoimmune disorders and allergies, which have increased in frequency in industrialized nations (10).

The study of the microbiology of the BE has expanded rapidly over the past several years, in part because of the rise of culture-independent methods for characterizing microbial diversity (4). Early work has found that humans rapidly transfer their microbes to BE surfaces (11–13), and these signatures can be used to forensically identify individuals (14–16). This transfer seems to be mainly unidirectional—i.e., surfaces look more human (microbiologically speaking) after an interaction rather than the other way around. Most of these microbes are likely dead, dying, or dormant—unable to grow on inert BE surfaces. Despite the inhospitable conditions on BE surfaces, many human-associated organisms can survive there for extended periods of time (17). Thus, BE surfaces are potential reservoirs of pathogenic and/or commensal organisms, although there is a paucity of evidence to show direct transfer of microbes from the BE to humans.

Much remains unknown about the relative importance of the different factors that shape the BE microbiome. Some work has been done to determine how the BE is influenced by direct or indirect dispersal from humans (11, 18), location within a room (12, 17, 19), surface material (13), environmental factors (20), and geography (3). However, these factors are often conflated with one another. Chase et al. (21) set out to control for these confounding variables in an office building experiment. They manufactured standardized sampling plates with sensors to detect temperature, humidity, and room occupancy. Each plate came with three sterilized BE surface types, i.e., painted drywall, ceiling tile, and carpet. Plates were installed on the floor, the wall, and the ceiling, at each of three office locations in three different cities (Flagstaff, AZ; San Diego, CA; and Toronto, ON). Office occupants were instructed not to touch the sampling plates, to prevent the direct transfer of human-associated microbes. Chase and colleagues found that the location of a plate (e.g., floor versus ceiling) and geography were the most important variables in shaping the BE microbial community composition. Surprisingly, the type of surface—drywall, tile, or carpet—had little impact on microbial community structure. Unlike prior work (11, 22), building-specific microbial signatures were weak when other factors were controlled for. The authors explain that this is likely due to the absence of direct contact between office workers and the sampling plates. Despite this lack of direct interaction, 25 to 30% of the BE surface microbial communities appeared to be from human skin. Overall, the authors suggest that BE surfaces are microbial deserts, wastelands like the Atacama Desert, where water and nutrients are scarce. They suggest that microorganisms from the human body or from environmental sources are dispersed onto these BE surfaces, where they either die or lie dormant, “waiting for liquid water to become active again.”

In addition to dissecting apart the BE determinants of microbial community composition, Chase and colleagues addressed two technical issues associated with BE research. First, they looked at how repeated sampling (dry swabbing) of the same surface perturbs the resident microbial community. They showed that multiple samplings of the same surface had a minimal effect on microbial community structure, opening the door to study designs that incorporate longitudinal sampling of the same surface. Second, they addressed the issue of batch effects (i.e., technical variation) between multiple sequencing runs by sequencing a subset of samples across all three of the runs completed in their study. These batch effects are especially pernicious for low-biomass samples, where PCR contaminants are much more common. Indeed, the authors detected a significant batch effect across runs and identified which taxa were differentially abundant. While the authors discuss strategies for mitigating this technical variance (e.g., filtering out taxa that differed significantly across runs), future work should focus on more refined methods for addressing these batch effects in high-throughput sequencing data. For example, batch effects are a known issue in microarray studies and strategies like factor analysis have been used successfully to remove this artificial variance from the data (23). Another, complementary, option would be to have a “gold standard” microbial community sample with a defined composition that researchers could include as a control in each sequencing run.

In summary, the study conducted by Chase and colleagues provides us with a better understanding of the basic rules governing community assembly on BE surfaces and the technical challenges associated with analyzing amplicon sequencing data. Unlike the lush habitats found in soils, lakes, oceans, and host organisms, BE surfaces appear to be barren wastelands. Microbial communities on BE surfaces are often the decaying remnants of the human microbiome. In the absence of direct human contact, microbes on BE surfaces are sourced from an ambient pool of airborne taxa. The persistence of microorganisms in the BE is the result of accumulation and dormancy and not active growth. If we want to reshape the BE microbiome, we need to control the rate of dispersal from different sources (e.g., environmental versus human). Epidemiological data suggest that the reduced microbial diversity found in BEs may have the largest impact on the developing microbiomes of infants and young children. Ultimately, the BE field will need to determine whether reduced exposure to microbial diversity in the BE is a major driver behind the hygiene hypothesis.
